# Enhanced Delivery of Thermoresponsive Polymer-Based Medicine into Tumors by Using Heat Produced from Gold Nanorods Irradiated with Near-Infrared Light

**DOI:** 10.3390/cancers13195005

**Published:** 2021-10-06

**Authors:** Kohei Sano, Yumi Ishida, Toshie Tanaka, Tatsuya Mizukami, Tomono Nagayama, Yoshie Haratake, Masayuki Munekane, Toshihide Yamasaki, Takahiro Mukai

**Affiliations:** Laboratory of Biophysical Chemistry, Kobe Pharmaceutical University, 4-19-1 Motoyamakita-machi, Higashinada-ku, Kobe 658-8558, Japan; yumi.ishida176@gmail.com (Y.I.); tw196037@st.kobepharma-u.ac.jp (T.T.); f15daisuki_0405@yahoo.co.jp (T.M.); fm162477@st.kobepharma-u.ac.jp (T.N.); bh196053@st.kobepharma-u.ac.jp (Y.H.); munekane@kobepharma-u.ac.jp (M.M.); yamasaki@kobepharma-u.ac.jp (T.Y.)

**Keywords:** thermoresponsive polymer, polyoxazoline, gold nanorod, near-infrared light, drug delivery system

## Abstract

**Simple Summary:**

To establish a therapy targeting scattered tumors throughout the body, we propose a novel drug delivery system using a thermoresponsive polyoxazoline (POZ) as a drug carrier in combination with gold nanorods (GNR), which produce heat when irradiated with near-infrared (NIR) light. After the tumor was irradiated with NIR light, where GNR was accumulated in advance, the radiolabeled POZ was intravenously injected. As a result, a marked tumor uptake was achieved via self-aggregation of POZ by sensing heat yielded from the GNR. Because the POZ would be chemically modified with various anti-tumor drugs including therapeutic radionuclides, remarkable anti-tumor effects can be expected by enhancing delivery of POZ-based medicine into scattered tumors throughout the body.

**Abstract:**

The aim of this study was to establish a drug delivery system (DDS) for marked therapy of tumors using a thermoresponsive polymer, polyoxazoline (POZ). The effectiveness of the following was investigated: (i) the delivery of gold nanorods (GNRs) to tumor tissues, (ii) heat production of GNR upon irradiation with near-infrared (NIR) light, and (iii) high accumulation of an intravenously injected radiolabeled POZ as a drug carrier in tumors by sensing heat produced by GNRs. When the GNR solution was irradiated with NIR light (808 nm), the solution temperature was increased both in a GNR-concentration-dependent manner and in a light-dose-dependent manner. POZ, with a lower critical solution temperature of 38 °C, was aggregated depending on the heat produced by the GNR irradiated by NIR light. When it was intratumorally pre-injected into colon26-tumor-bearing mice, followed by NIR light irradiation (GNR+/Light+ group), the tumor surface temperature increased to approximately 42 °C within 5 min. Fifteen minutes after irradiation with NIR light, indium-111 (^111^In)-labeled POZ was intravenously injected into tumor-bearing mice, and the radioactivity distribution was evaluated. The accumulation of POZ in the tumor was significantly (approximately 4-fold) higher than that in the control groups (GNR+/without NIR light irradiation (Light–), without injection of GNR (GNR–)/Light+, and GNR–/Light– groups). Furthermore, an in vivo confocal fluorescence microscopy study, using fluorescence-labeled POZ, revealed that uptake of POZ by the tumor could be attributed to the heat produced by GNR. In conclusion, we successfully established a novel DDS in which POZ could be efficiently delivered into tumors by using the heat produced by GNR irradiated with NIR light.

## 1. Introduction

Since side effects can still be a major concern in cancer therapy, cancer-selective drug delivery is essential for superior therapy without incurring systemic toxicities. Presently, nanocarriers such as liposomes and micelles have been employed to deliver more drugs per carrier (nanoparticle) into tumor tissues via the enhanced permeability and retention (EPR) effect [1,2] in pre-clinical and clinical stages [3,4,5,6,7]. More recently, water-soluble polymers have attracted considerable attention in the field of drug delivery systems for diagnosis and therapy (theranostics) because of their biocompatibility, availability of a wide range of molecular masses, and facile modification of polymer chains. To date, some reports have revealed that water-soluble polymers, including polyethylene glycol (PEG) [8], *N*-(2-hydroxypropyl)methacrylamide (HPMA) [9], polysarcosine [10], and polyoxazoline (POZ) [11,12,13], could accumulate in the tumor via passive and active tumor-targeting mechanisms.

In particular, polyoxazoline (POZ) has a lower critical solution temperature (LCST) and is used in drug delivery systems (DDS) as a thermoresponsive polymer [14]. We propose a novel tumor therapy where a POZ as a drug carrier could be delivered into tumors via thermoresponsive aggregation of POZ in combination with tumor-localized hyperthermia [15]. In fact, when the radiolabeled POZ was intravenously injected, an approximate two-fold increase in drug delivery was observed in heat-treated tumors compared to non-heat-treated tumors. However, this strategy using hyperthermia could be applied only to tumors identified and localized before treatment.

Gold nanorods (GNRs), characterized by a uniform shape with a narrow size distribution, are anisotropic gold nanoparticles because of their two absorption peaks on the basis of longitudinal and transversal plasmon resonances [16]. Their optical and chemical properties can be altered based on shape and aspect ratio (longitude/transverse), leading to various biomedical applications [17,18]. Irradiation of near-infrared (NIR) light into GNRs could yield a moderate temperature rise, causing damage to the target tumor tissue, which is more sensitive to hyperthermia than healthy tissue. Therefore, photothermal tumor therapies using GNRs have been intensively investigated owing to their excellent photothermal conversion efficiency [19,20,21,22]. In contrast to conventional localized hyperthermia, tumor-selective heat production can be achieved throughout the body by using GNRs conjugated with tumor-targeting moieties.

**Figure 1:**
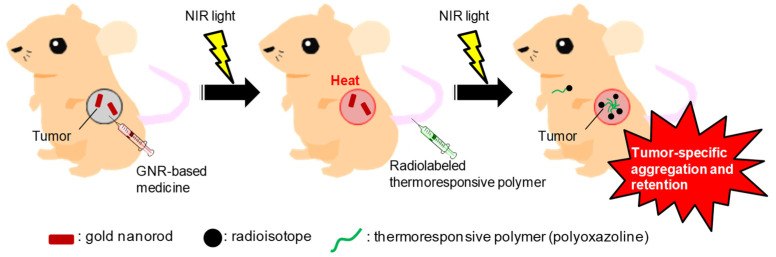
Tested novel drug delivery system using a radiolabeled thermoresponsive polyoxazoline that is self-aggregating in tumor tissue when given gold nanorods (GNRs) and irradiated with near-infrared (NIR) light.

In this study, to establish a therapy targeting systemic tumors, we proposed a novel drug delivery system as follows (Figure 1): (i) GNRs (heat source) is delivered into the tumors, (ii) the intratumoral temperature is increased by irradiation by NIR light, and (iii) POZ intravenously injected as a drug carrier can be efficiently taken up by the tumors via polymer aggregation. In this study, the GNR was intratumorally injected in step (i) to validate the tumor uptake of thermoresponsive POZ dependent on the heat produced by GNRs as a proof-of-concept study. The radiolabeled POZ was used to evaluate the distribution of POZ throughout the body quantitatively.

## 2. Materials and Methods

### 2.1. Optical and Chemical Properties of GNR

GNRs functionalized by carboxylic acid (size; 10 × 45 nm, longitudinal surface plasmon resonance; 808 nm, 94 nM) was purchased from Nanopartz Inc. (Loveland, CO, USA). We actually measured the size (Z-average fractioned mean diameters) and zeta potential in PBS using a Zetasizer Nano (Malvern Instruments Ltd., Malvern, UK). Furthermore, we evaluated the photostability of GNR (1 nM in PBS) by irradiating NIR light (SPOLD, Hamamatsu Photonics, Hamamatsu, Japan) of 0.6 W/cm^2^ for 0, 5, 10, and 15 min based on the Vis-NIR absorbance spectra.

### 2.2. Evaluation on Temperature Change of GNR Solution upon NIR Light Irradiation

The concentration of GNR was adjusted to 0, 0.1, 0.25, 0.5, and 1.0 nM by dilution with distilled water. The GNR samples were irradiated with NIR light (808 nm, 1, 2, or 5 W/cm^2^) for 5 min, and the solution temperature was monitored every 15 s using a thermographic camera (FLIR E4, FLIR Systems, Inc., Wilsonville, OR, USA).

### 2.3. Aggregation of POZ via Heat Yielded from GNRs by NIR Light Irradiation

**Figure 2:**
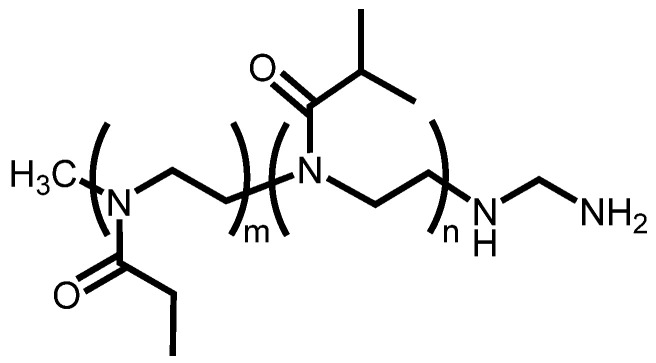
Chemical structure of POZ derivative investigated in this study.

A POZ derivative was synthesized as described in our previous report [23]. Its molecular structure in this study is shown in Figure 2. ^1^H NMR spectrum for the POZ derivative was recorded by JEOL JNM-ECZ400S (400 MHz) (Figure S1). The molecular weight of POZ was 22,342 g/mol, as measured by gel permeation chromatography. The LCST of POZ was 38 °C, which was determined using a Zetasizer Nano. The hydrated size of POZ was measured at 37 and 42 °C in phosphate-buffered saline (PBS). The POZ solution (100 μM, 100 μL PBS) was mixed with GNRs (0 or 1 nM, 100 μL PBS) in the tubes. The tubes were irradiated with NIR light (2 W/cm^2^) for 5 min, and then the aggregation of POZ was observed.

### 2.4. Synthesis of ^111^In-Labeled POZ and Fluorescence-Labeled POZ

^111^In-Labeling of POZ was performed according to our previous report [15]. ^111^InCl_3_ was kindly provided by Nihon Medi-Physics (Tokyo, Japan). After preparation of POZ (LCST: 38 °C, M.W.: 21,645) modified by *p*-SCN-Bn-DOTA (Macrocyclics Inc., Dallas, TX, USA) (DOTA-POZ), ^111^InCl_3_ was reacted with DOTA-POZ in acetate buffer (0.1 M, pH 6.0) at room temperature for 30 min. After the addition of excess EDTA (50 equiv.), purification with Amicon Ultra-4 (molecular weight cut-off (MWCO): 3 kDa, 7500× *g*) (Merck Millipore, Co., Billerica, MA, USA) was conducted by elution with PBS. The radiochemical purity was calculated using a PD-10 column (GE Healthcare, Piscataway, NJ, USA).

Furthermore, fluorescence-labeled POZ was synthesized to evaluate the intratumoral distribution of POZ by confocal fluorescence microscopy. POZ (5.45 mg, 1 eq.) was mixed with Fluorescein-5-isothiocyanate (FITC) (Life Technologies Co., Carlsbad, CA, USA) (0.49 mg, 5 eq.) in PB (0.1 M, pH 9.0) (500 μL), and then stirred at room temperature for 24 h. After the reaction, the mixture was diafiltrated twice with Amicon Ultra-4 (MWCO: 3 kDa) using distilled water as a solvent to remove unreacted FITC, and the purified sample was freeze-dried for storage. The number of FITC molecules conjugated to POZ was calculated by measuring absorbance at 488 nm, as reported previously [15].

### 2.5. Cell Culture and Animal Model

Colon26, a mouse cell line derived from rectal cancer, was purchased from the RIKEN Bio Resource Center, Japan. The cells were harvested in DMEM (Life Technologies Co.) containing 10% fetal bovine serum, 100 units/mL penicillin, and 100 μg/mL streptomycin in 5% CO_2_ at 37 °C. Animal experiments were performed according to institutional guidelines. The protocol was approved by the Kobe Pharmaceutical University Committee for Animal Care and Use. Colon26 cells (1 × 10^6^ cells) were inoculated in both the right and left flanks of BALB/c mice (male, 5 weeks old) after local hair removal, and the mice were used for biodistribution study 7–8 days after inoculation. During the procedure, mice were anesthetized with isoflurane.

### 2.6. Biodistribution of ^111^In-Labeled POZ When Combined with GNR-Based Hyperthermia

The GNR solution or PBS (5 μL) was intratumorally injected into the tumors in both the right and left flanks of mice. The GNR concentration was adjusted to 1 nM. One side was irradiated with NIR light (0.6 W/cm^2^) for 15 min, and the other side was shielded from light using aluminum foil. The change in tumor surface temperature was monitored using thermography (FLIR E4). While continuing light irradiation, ^111^In-labeled POZ (4 nmol/100 μL) was intravenously administered, and the mice were sacrificed 60 min after probe injection. The blood, spleen, pancreas, stomach, intestine, kidney, liver, heart, lung, muscle, and tumor were excised, and their weights were measured. The radioactivity of these organs was determined using a gamma counter (Wizard2480, PerkinElmer, Inc., Waltham, MA, USA). The accumulation of radioactivity in these organs was represented as the percentage injected dose (%ID)/g of tissue.

### 2.7. Fibered Confocal Fluorescence Microscopic Imaging Studies

The intratumoral localization of POZ was investigated using a fibered confocal fluorescence microscopy imaging system (Cellvizio^®^ Endomicroscopy System, Mauna Kea Technologies, Paris, France) with an S-1500 probe (field of view: 600 μm diameter, lateral resolution: 3.3 μm). By combining GNR injection with NIR light irradiation, the tumors were pre-heated for 15 min as mentioned above. Thereafter, FITC-labeled POZ (34 nmol/150 μL/mouse) was intravenously injected. The tumor temperature was maintained at 42–43 °C for an additional 60 min, followed by confocal fluorescence microscopy. The imaging data were acquired at a scan rate of 12 frames/s.

### 2.8. Statistical Analysis

Four to six mice were used for in vivo experiments. Statistical significance between groups was determined using Tukey’s test. Data were presented as the mean ± standard deviation. Statistical significance was set at *p* < 0.05.

## 3. Results

### 3.1. Optical and Chemical Properties of GNR

The size and zeta-potential of GNR were 17.0 ± 3.3 nm and −10.5 ± 1.5 mV, respectively. GNR used in this study has an absorbance peak at around 800 nm, and was highly stable after the irradiation of NIR light (Figure S2). The biocompatibility of GNR with carboxyl groups was reported by Wang et al. [24].

### 3.2. Evaluation on Temperature Change of GNR Solution upon NIR Light Irradiation

**Figure 3:**
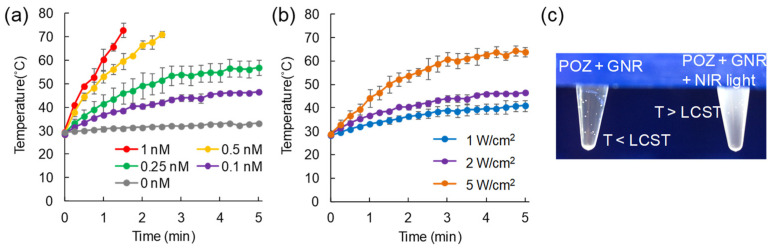
Photothermal effect of gold nanorods (GNRs) irradiated with near-infrared (NIR) light. (**a**) Temperature elevation over a period of 5 min of NIR light irradiation (2 W/cm^2^) at different GNR concentrations (0–1 nM). (**b**) The effect of NIR light dose (1–5 W/cm^2^) on temperature elevation (GNR concentration: 0.1 nM). (**c**) Aggregation of polyoxazoline (POZ) via heat yielded from GNR by NIR light irradiation.

The change in the temperature of the GNR solutions was evaluated by thermography when irradiated with NIR light (Figure 3). The temperature of the solutions was increased by irradiation with NIR light (2 W/cm^2^) in a GNR-concentration-dependent manner (Figure 3a). Furthermore, a higher light dose enhanced the increase in the GNR solution temperature (Figure 3b).

### 3.3. Aggregation of POZ via Heat Yielded from GNR by NIR Light Irradiation

The aggregation of POZ was evaluated in mixed solutions of GNR and POZ upon light irradiation. Immediately upon light irradiation, white turbidity of the POZ solution was observed, suggesting the aggregation of POZ via an increase in solution temperature (Figure 3c). On the other hand, POZ was not aggregated in the solution without light irradiation, regardless of coexistence with GNR. The hydrated size of POZ was 4.7 ± 1.8 nm and 429 ± 138 nm at 37 °C and 42 °C, respectively (Figure S3).

### 3.4. Synthesis of FITC-Labeled POZ and ^111^In-Labeled POZ

In the chemical structure of the POZ derivative (Figure 2), the m:n was approximately 3:1, which was determined by analysis of NMR data (Figure S1). The number of FITC molecules conjugated to POZ was approximately 0.5, as determined by the absorbance at 488 nm. ^111^In-labeled POZ derivatives were successfully prepared with a radiochemical yield of 56.2% and a radiochemical purity greater than 94%, which was determined using a gel filtration column.

### 3.5. Evaluation of Distribution of ^111^In-Labeled POZ by Combination with GNR-Based Hyperthermia

**Figure 4:**
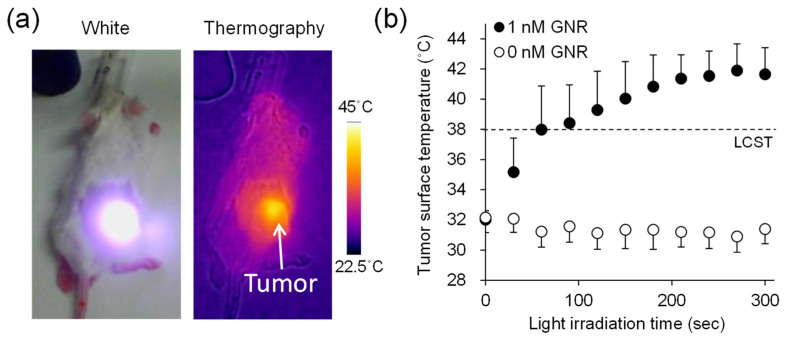
Change in tumor surface temperature under near-infrared (NIR) light irradiation to the tumor where gold nanorods (GNRs) were highly taken up. (**a**) Thermographic images. (**b**) Change in tumor surface temperature after irradiation. LCST: lower critical solution temperature.

Under isoflurane anesthesia, tumor tissues were irradiated with NIR light (0.6 W/cm^2^, 15 min), where GNR solutions were intratumorally injected (intratumoral GNR concentration: 1 nM). Immediately (~150 s) after light irradiation, the tumor surface temperature increased to 40–42 °C (above the lower critical solution temperature (LCST)) (Figure 4), which was maintained for 60 min under light irradiation. In contrast, there was no marked change in the tumor surface temperature (31–32 °C) under the condition of GNR +/NIR light−.

**Figure 5:**
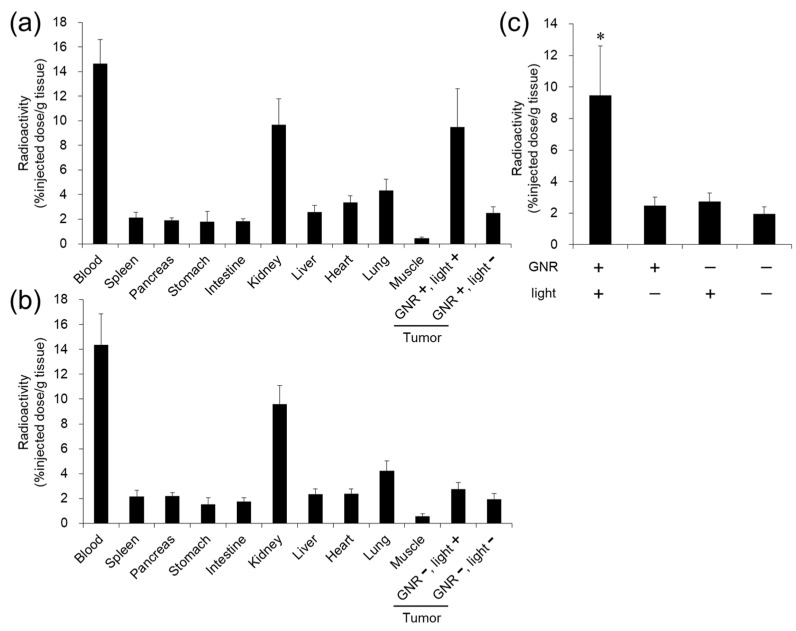
Biodistribution of ^111^In-labeled polyoxazoline (POZ) by combination with hyperthermia using gold nanorods (GNRs) irradiated by near-infrared (NIR) light. (**a**) GNR was injected into both tumors inoculated in right and left flanks, and then NIR light was irradiated into the one side of tumors. (**b**) Phosphate-buffered saline (PBS) was injected into both tumors in right and left flanks, and then NIR light irradiated a tumor on one side. (**c**) Radioactivity accumulation in the tumor treated with or without GNR injection and NIR light irradiation. * *p* < 0.001 vs. other groups.

Fifteen minutes after NIR light irradiation, ^111^In-labeled POZ was intravenously injected, and the distribution in the tumor-bearing mice was evaluated (Figure 5). High radioactivity was detected in the tumor where GNR had been intratumorally injected followed by NIR light irradiation (GNR+/Light+ group), 1 h post-injection of probes (9.5 ± 3.1% injected dose per gram (ID/g). On the other hand, the accumulation of radioactivity in the tumor was 2.5 ± 0.5, 2.8 ± 0.5, and 2.0 ± 0.5% ID/g for GNR+/Light−, GNR−/Light+, and GNR−/Light− groups, respectively. A significantly higher (approximately 4-fold) tumor uptake of ^111^In-labeled POZ was observed in the GNR+/Light+ group than in the other groups, suggesting that the tumor uptake of POZ was caused by heat produced from GNR. Low levels of radioactivity were observed in normal tissues, including the lungs (4.3 ± 0.8% ID/g), liver (2.5 ± 0.5% ID/g), and spleen (2.1 ± 0.4% ID/g), except for the blood (14.5 ± 2.0% ID/g) and kidneys (9.7 ± 1.8% ID/g) (average values collected from all mice).

### 3.6. Fibered Confocal Fluorescence Microscopic Imaging Studies

**Figure 6:**
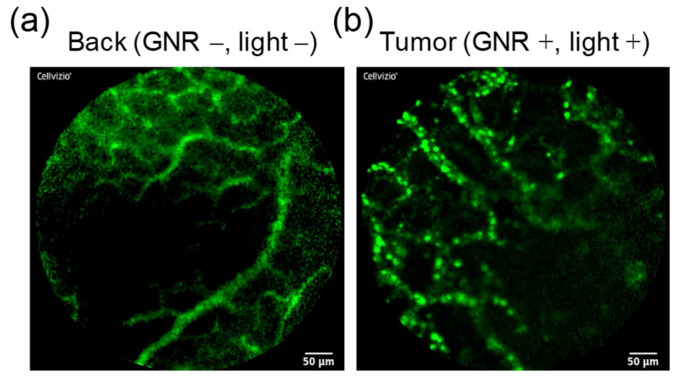
Distribution of fluorescence-labeled polyoxazoline (POZ) in the untreated normal tissue (back) (**a**) and in the tumor treated with gold nanorods (GNRs) and near-infrared (NIR) irradiation (**b**), which was visualized using confocal microscopy. Green: FITC-labeled POZ. Scale bar = 50 μm.

Furthermore, the intratumoral distribution of FITC-labeled POZ was evaluated using a fibered confocal fluorescence microscope (Figure 6, Movies S1 and S2). In the tumor injected with GNRs and irradiated with NIR light, the distribution of FITC–POZ was localized near the blood vessels (Movie S1). In contrast, in the untreated normal tissue (back), fluorescence signals derived from FITC–POZ were observed mainly within the blood vessels (Movie S2).

## 4. Discussion

POZ is a biocompatible water-soluble polymer and is expected to be used in the biomedical field owing to its unique characteristics. This study demonstrated that the tumor uptake of radiolabeled POZ (LCST: 38 °C) was markedly enhanced when combined with GNR administration and irradiation of NIR light via POZ self-aggregation by sensing heat produced by GNR. The tumor uptake of POZ was approximately 10% ID/g, which was similar to that of superior antibody-based radiopharmaceuticals targeting tumor-specific antigens, although it depends on the conditions including the tumor cell line, radionuclide, injection dose, and evaluation timing [25]. Furthermore, in traditional hyperthermia, the treatment is generally given for 30–60 min [26]. Photothermal therapy using GNRs has also been used within 1 h in many reports [19,20]. In this study, polymer aggregation was successfully induced early after treatment, and as a result, the amount of POZ delivered to the tumor was dramatically improved within 1.25 h. (For the dosage and administration timing of the POZ, we referred to our previous paper [15]). These results suggested that it is easy to incorporate the treatment of POZ-based medicine into clinically implemented protocols. Kawano et al. reported a photothermal therapy using GNR modified with thermoresponsive water-soluble polymer on their surface, and the amount of GNRs delivered to the tumor improved in combination with light irradiation [27]. We proposed the more versatile therapeutic strategy where the delivery of thermoresponsive POZ as a drug carrier into the tumor could be remarkably improved by sensing heat from the GNRs.

Recently, therapeutic radiopharmaceuticals have attracted much attention owing to their extraordinary use in internal radiotherapy of tumors. For instance, prostate-specific membrane antigen-targeted radiopharmaceuticals labeled with various β^−^ and α-ray emitting radionuclides, including yttrium-90 [28], lutetium-177 (Lu-177) [29], and actinium-225 [30] demonstrated marked therapeutic effects. Furthermore, a Lu-177-labeled peptide-based radiopharmaceutical (Lu-177-dotatate) targeting somatostatin receptor 2, which is highly expressed in neuroendocrine tumors, was approved by the Food and Drug Administration [31]. POZ–DOTA could also be labeled with these therapeutic radiometals as previously reported [23]; therefore, the application of this strategy using a radiolabeled thermoresponsive POZ would be expected for efficient internal radiotherapy of tumors.

The most important issue in this strategy is being able to control the systemic distribution of GNRs precisely. The pharmacokinetics of GNRs have been thoroughly investigated, and Akiyama et al. reported that PEG-modified GNRs are mainly trapped in the liver and spleen, suggesting clearance by the reticuloendothelial system [32]. The relatively higher tumor uptake via the EPR effect has been reported compared to that in the blood, kidney, and lung. Furthermore, in recent years, GNR-based medicines conjugated with tumor-targeting moieties have been energetically studied [33,34], and antibody-GNR conjugates have dramatically improved the tumor accumulation of GNRs [33].

Although it is essential to shield organs (liver and spleen) from non-specific accumulation from light irradiation, the temperature increase in the tumor would be relatively high when the NIR light irradiation is carried out systematically, and the proposed DDS would work well. Notably, a strategy to maintain the temperature of normal organs below the LCST of POZ would also be required to reduce unnecessary side effects. We believe that this therapeutic strategy would cure scattered tumors that cannot be treated by local hyperthermia.

## 5. Conclusions

In this study, we revealed that the radiolabeled thermoresponsive POZ as a drug carrier could be markedly taken up by the tumor when it was irradiated by NIR light where GNRs had highly accumulated. Therefore, we successfully established a novel DDS in which POZ could be efficiently delivered into tumors using a light-responsive GNR.

## Data Availability

The data presented in this study are available within the article or Supplementary Materials.

## Supplementary Materials

The following are available online at https://www.mdpi.com/article/10.3390/cancers13195005/s1, Figure S1: ^1^H NMR spectrum of a POZ derivative investigated in this study, Figure S2: Vis-NIR spectra of GNR investigated in this study. At 0, 5, 10, and 15 min after irradiation of NIR light (0.6 W/cm^2^), the Vis-NIR spectra was evaluated, Figure S3: Size distribution of POZ derivative investigated in this study in PBS at 37 °C (A) and 42 °C (B), determined using dynamic light scattering measurements. The number-average diameters were shown, Movie S1: Confocal microscopy imaging of POZ distribution in the tumor tissues treated with gold nanorod (GNR) administration and near-infrared (NIR) light irradiation, Movie S2: Confocal microscopy imaging of POZ distribution in the non-treated normal tissues.
