# Supplementary material for: Enhanced Delivery of Thermoresponsive Polymer-Based Medicine into Tumors by Using Heat Produced from Gold Nanorods Irradiated with Near-Infrared Light
Source: Cancers (Basel). 2021 Oct 6;13(19):5005. doi: 10.3390/cancers13195005 (PMC8508138; doi:10.3390/cancers13195005)
Supplement: Supplementary file 1 [file cancers-13-05005-s001.zip › 210930-revise2-210830_Supplementary materials.pdf]

cancers

Supplementary Materials

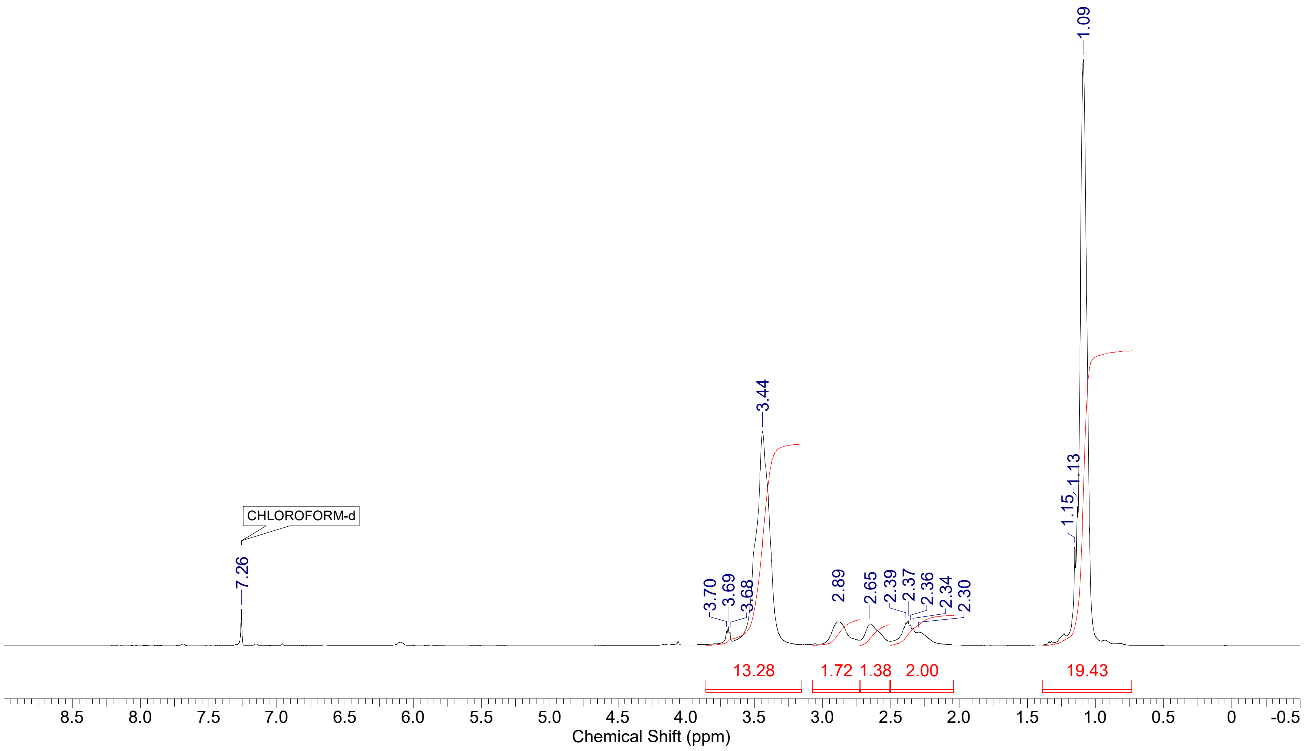

Figure S1. ^{1}**H NMR spectrum of a POZ derivative investigated in this study.**

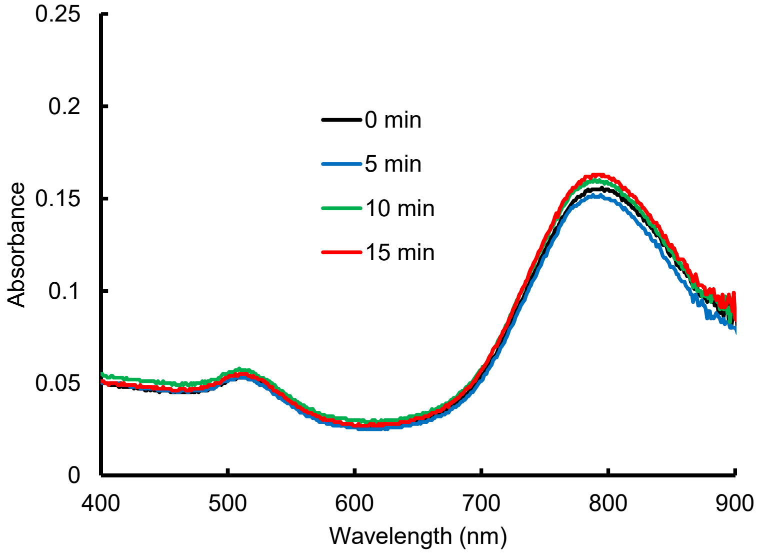

Figure S2. Vis-NIR spectra of GNR investigated in this study. At 0, 5, 10, and 15 min after irradiation of NIR light (0.6 W/cm²), the Vis-NIR spectra was evaluated.

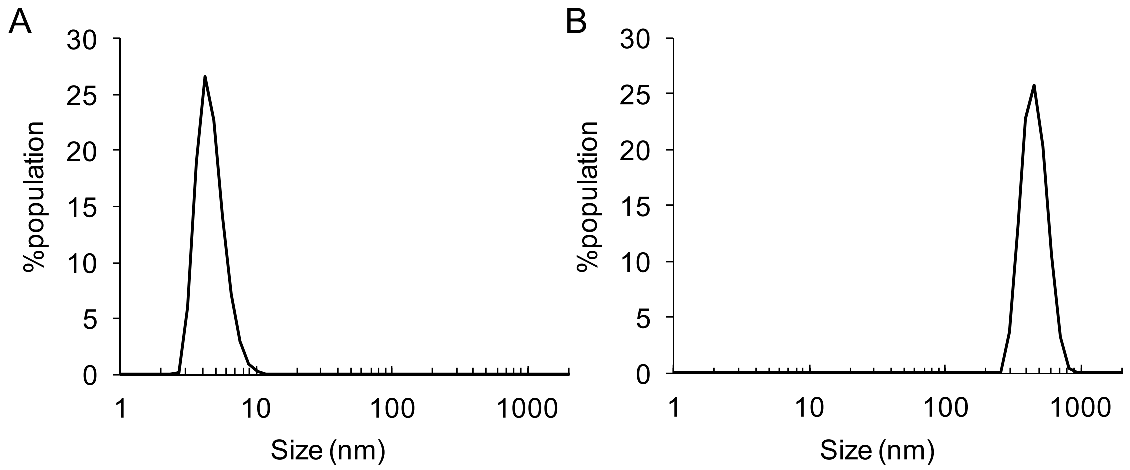

Figure S3. Size distribution of POZ derivative investigated in this study in PBS at 37 ˚C (A) and 42 ˚C (B), determined using dynamic light scattering measurements. The number-average diameters were shown.

Movie S1. Polyoxazoline (POZ) distribution in tumor tissues treated with gold nanorod (GNR) administration and near-infrared (NIR) light irradiation was observed in real-time. Confocal fluorescence microscopy imaging was performed 60 min after probe injection. Green: FITC-labeled POZ. Scale bar=50 µm.

Movie S2. Polyoxazoline (POZ) distribution in untreated normal tissues (back) observed in real-time. Confocal fluorescence microscopy imaging was performed 60 min after probe injection. Green: FITC-labeled POZ. Scale bar=50 µm.
